# Supplementary material for: Quantitative pathogenicity and host adaptation in a fungal plant pathogen revealed by whole-genome sequencing
Source: Nat Commun. 2024 Mar 2;15:1933. doi: 10.1038/s41467-024-46191-1 (PMC10908820; doi:10.1038/s41467-024-46191-1)
Supplement: Supplementary file 1 — Supplementary Information [file 41467_2024_46191_MOESM1_ESM.pdf]

Supplementary Information

Quantitative pathogenicity and host adaptation in a fungal plant pathogen revealed by whole-genome sequencing

Reda AMEZROU, Aurélie DUCASSE, Jérôme COMPAIN, Nicolas LAPALU, Anais PITARCH, Laetitia DUPONT, Johann CONFAIS, Henriette GOYEAU, Gert HJ KEMA, Daniel CROLL, Joëlle AMSELEM, Andrea SANCHEZ-VALLET, Thierry C MARCEL

Correspondence: T.C.M. (thierry.marcel@inrae.fr) and R.A. (reddamez@gmail.com)

Supplementary Figures

Supplementary Fig. 1 | Percentage of genetic variance explained by principal component analysis.
Supplementary Fig. 2 | Heatmap on the kinship matrix calculated with the full SNP matrix using VanRaden method.
Supplementary Fig. 3 | Phenotypic distribution of PLACP and PLACN quantitative pathogenicity traits divided over cultivars from which isolates originated.
Supplementary Fig. 4 | Quantile-quantile plots of multi-locus mixed linear model (pink curve) and single-locus linear model (blue curve) for the different trait-cultivar associations.
Supplementary Fig. 5 | PLACP of individual *Zt_6_00682* mutants complemented with the virulent allele (grey boxes) and the avirulent allele (white boxes) in the *Zt_6_00682* knockout background.
Supplementary Fig. 6 | PLACP produced by the wild-type strain 3D7, *Zt_6_682* knockout mutants and the complementation mutants carrying the virulent (3D7) and avirulent (3D1) allele in different cultivars.
Supplementary Fig. 7 | PLACP of individual *Zt_3_00467* mutants on the cultivar 'Arina' (*Stb6* and *Stb15*) expressing the avirulent allele (white boxes), the virulent allele (green boxes) and the wild-type strain 3D7.
Supplementary Fig. 8 | PLACP of *Zt_3_00467* mutants on the cultivar 'Riband' (*Stb15*) expressing the avirulent allele (green box), the virulent allele (blue box) and the wild-type strain 3D7.
Supplementary Fig. 9 | Phylogenetic tree of *Zt_3_00467* and *Zt_6_00682* generated from the protein sequences of the global *Z. tritici* isolates (n=241 and 248, respectively).

Supplementary Tables

Supplementary Table 1 | Descriptive statistics for quantitative pathogenicity traits assessed on differential wheat cultivars.
Supplementary Table 2 | Analysis of variance summary table showing the influence of isolate, cultivar and their interaction.
Supplementary Table 3 | Wheat differentials used to pathotype *Z. tritici* isolates and their known Stb resistance genes indicated in grey (Brown et al., 2015).
Supplementary Table 4 | Functional annotation of candidate pathogenicity genes.
Supplementary Table 5 | Diversity statistics of pathogenicity genes.
Supplementary Table 6 | Summary of recombination and selection analyses of pathogenicity genes.
Supplementary Table 7 | List of primers used for functional studies.

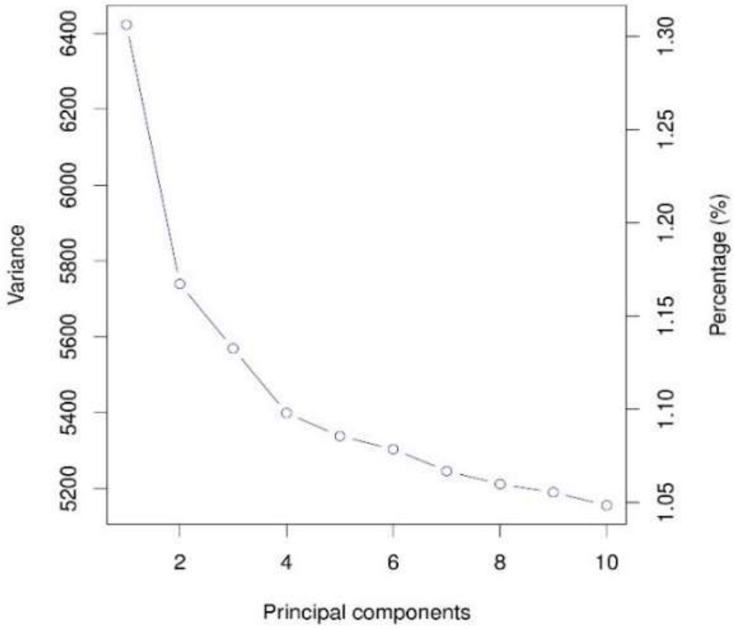

Supplementary Fig. 1 | Percentage of genetic variance explained by principal component analysis.

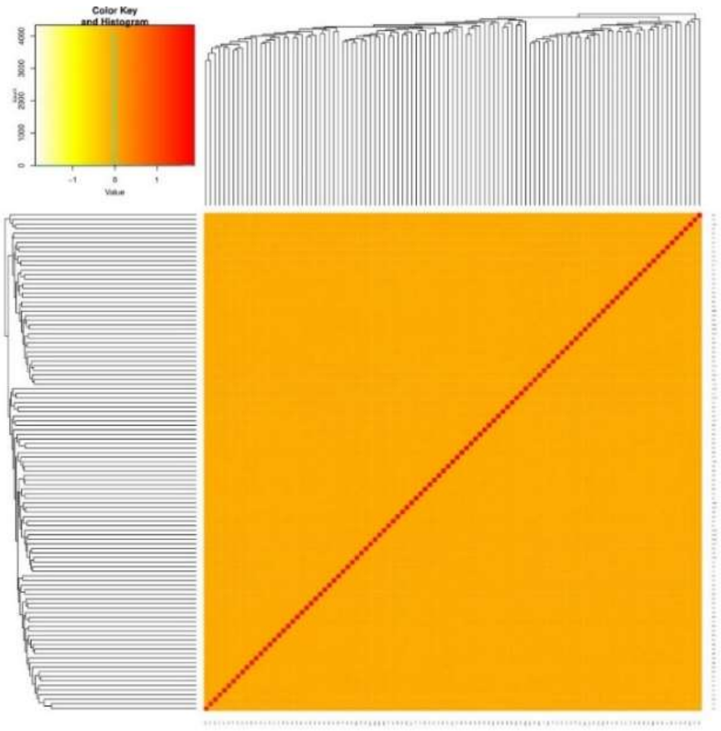

Supplementary Fig. 2 | Heatmap on the kinship matrix calculated with the full SNP matrix using VanRaden method.

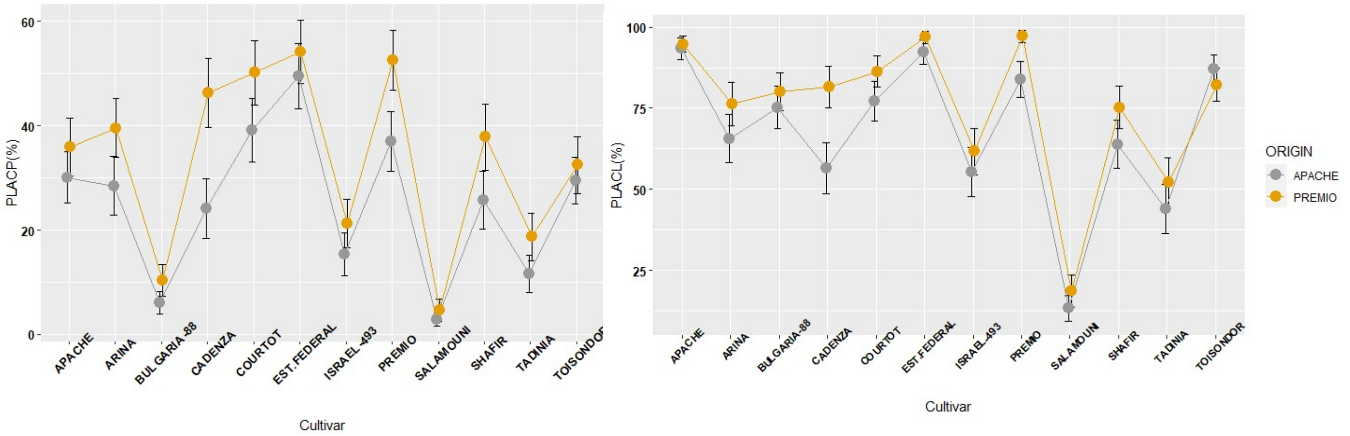

Supplementary Fig. 3 | Phenotypic distribution of PLACP and PLACN quantitative pathogenicity traits divided over cultivars from which isolates originated.

PLACP

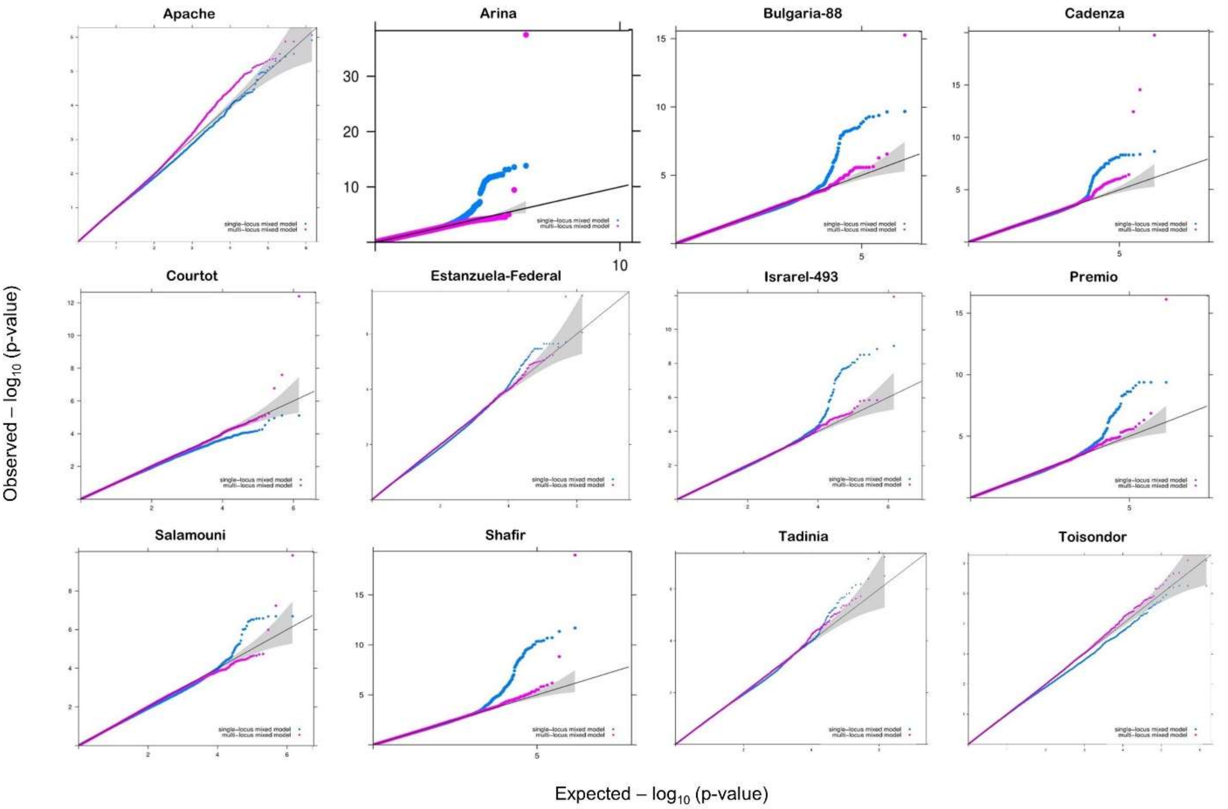

PLACN

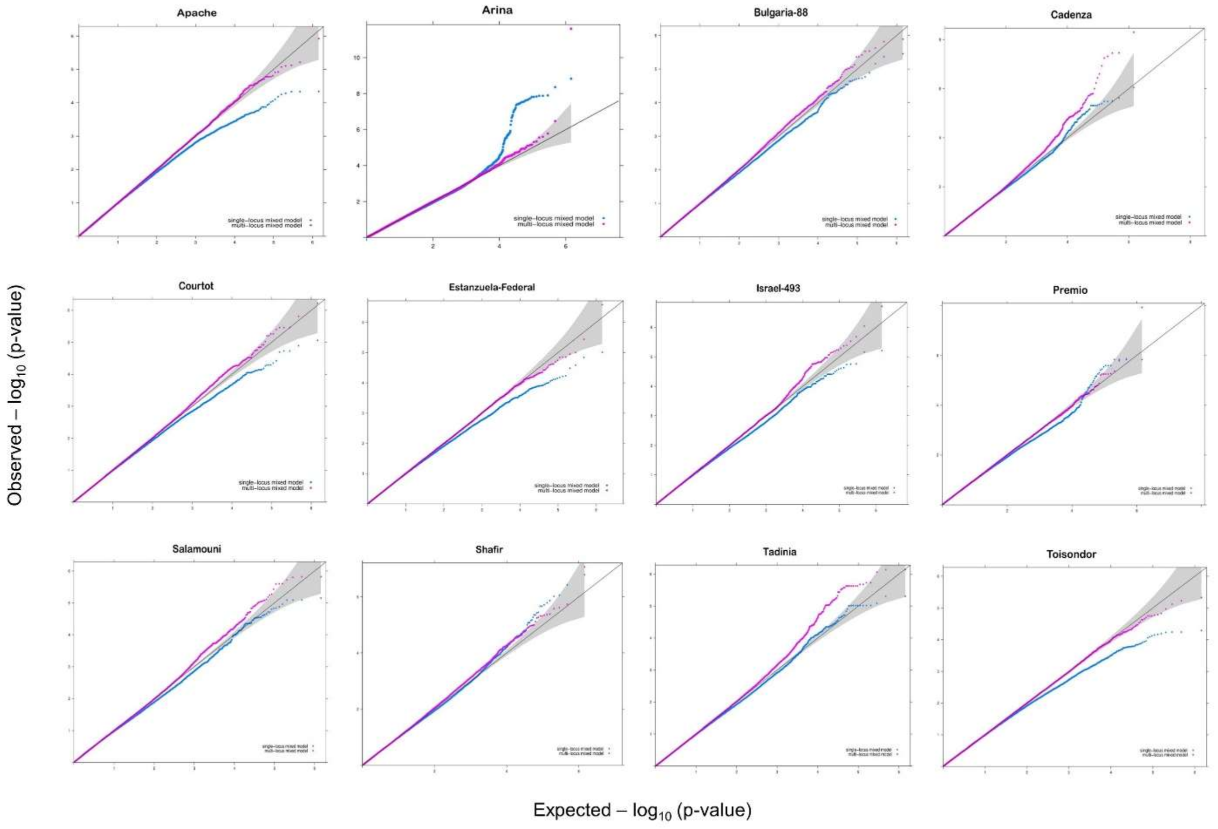

Supplementary Fig. 4 | Quantile-quantile plots of multi-locus mixed linear model (pink curve) and single-locus linear model (blue curve) for the different trait-cultivar associations.

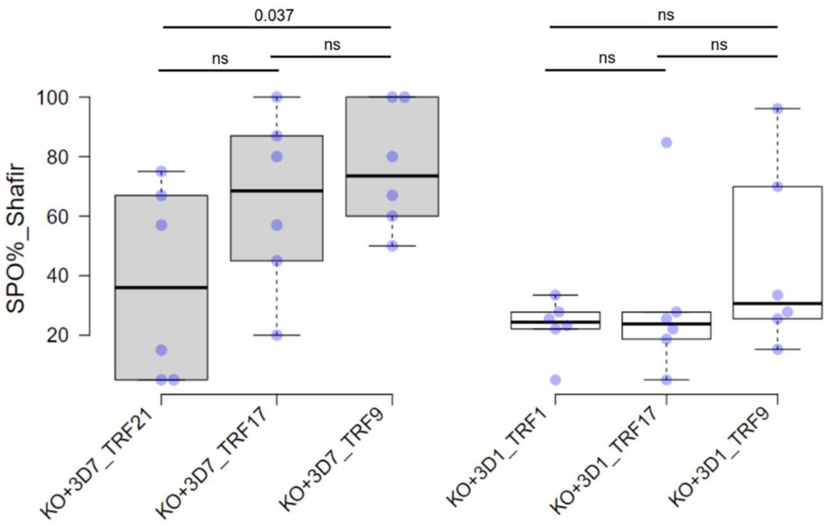

Supplementary Fig. 5 | PLACP of individual *Zt_6_00682* mutants complemented with the virulent allele (grey boxes) and the avirulent allele (white boxes) in the *Zt_6_00682* knockout background. Significance was determined with Wilcoxon rank-sum test. Data are presented as box plots (centre line at the median, upper bound at 75th percentile, lower bound at 25th percentile) with whiskers chosen to show the 1.5 of the interquartile range. Each dot represents one inoculated leaf.

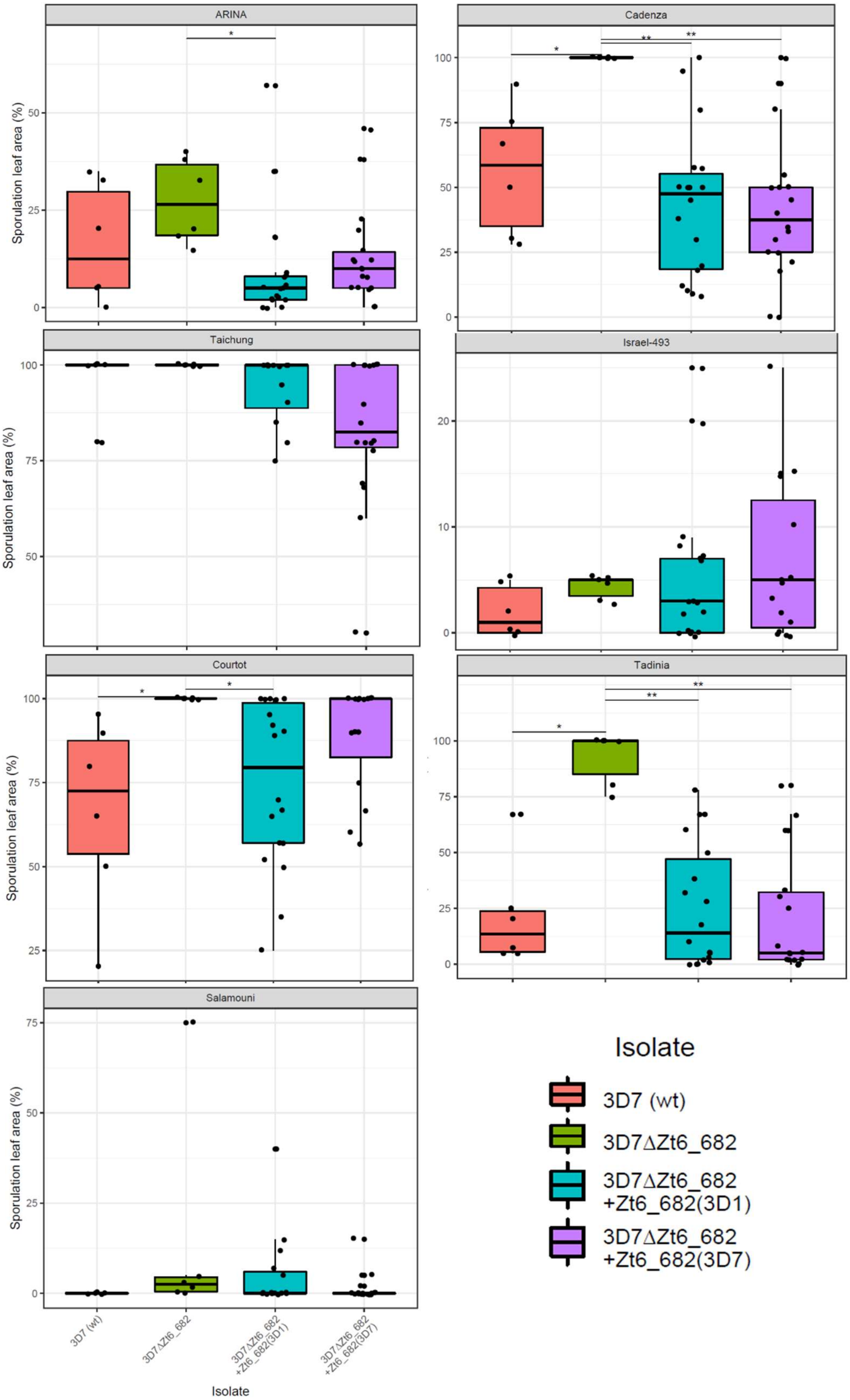

Supplementary Fig. 6 | PLACP produced by the wild-type strain 3D7, *Zt_6_00682* knockout mutants and the complementation mutants carrying the virulent (3D7) and avirulent (3D1) allele in different cultivars. KO mutants were highly aggressive in the cultivars 'Cadenza', 'Tadinia' and 'Courtot'. Significance was determined with Wilcoxon rank-sum test ($P<0.05$*; $P<0.01$**). Data are presented as box plots (centre line at the median, upper bound at 75th percentile, lower bound at 25th percentile) with whiskers chosen to show the 1.5 of the interquartile range. Each dot represents one inoculated leaf.

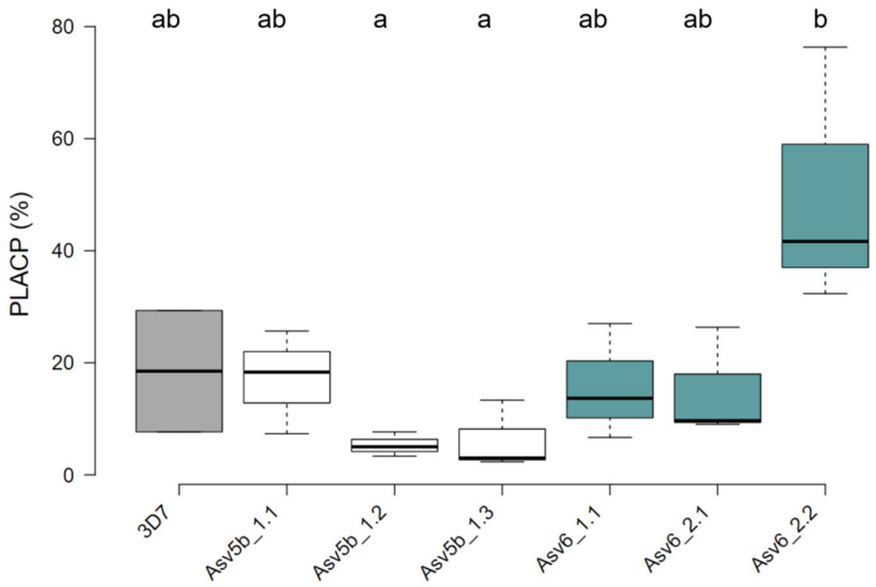

Supplementary Fig. 7 | PLACP of individual *Zt_3_00467* mutants on the cultivar 'Arina' (Stb6 and Stb15) expressing the avirulent allele (white boxes), the virulent allele (green boxes) and the wild-type strain 3D7. Statistical significance was determined using a Tukey's HSD test. Data are presented as box plots (centre line at the median, upper bound at 75th percentile, lower bound at 25th percentile) with whiskers chosen to show the 1.5 of the interquartile range.

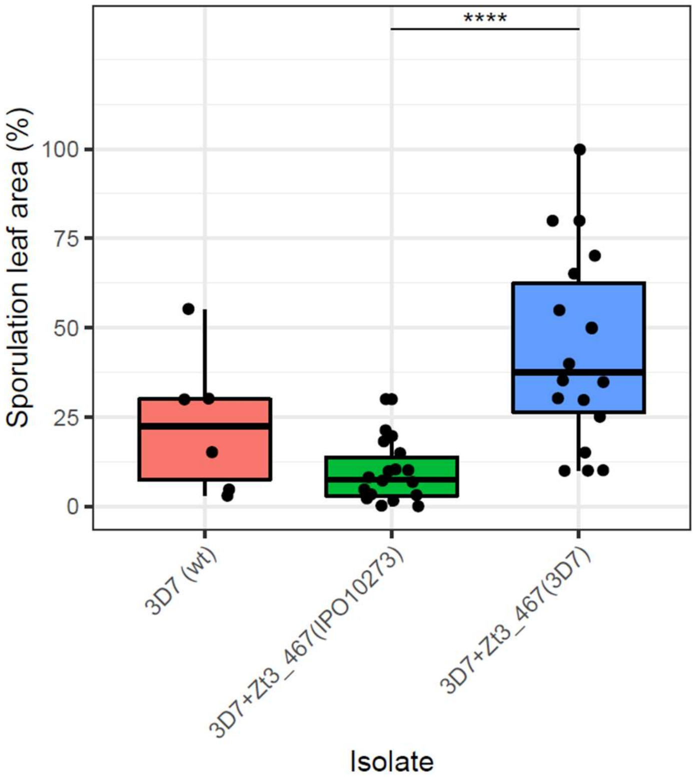

Supplementary Fig. 8 | PLACP of *Zt_3_00467* mutants on the cultivar 'Riband' (Stb15) expressing the avirulent allele (green box), the virulent allele (blue box) and the wild-type strain 3D7. Significance was determined with Wilcoxon rank-sum test (*P*<0.0001****). Data are presented as box plots (centre line at the median, upper bound at 75th percentile, lower bound at 25th percentile) with whiskers chosen to show the 1.5 of the interquartile range. Each dot represents one inoculated leaf.

Zt_3_00467

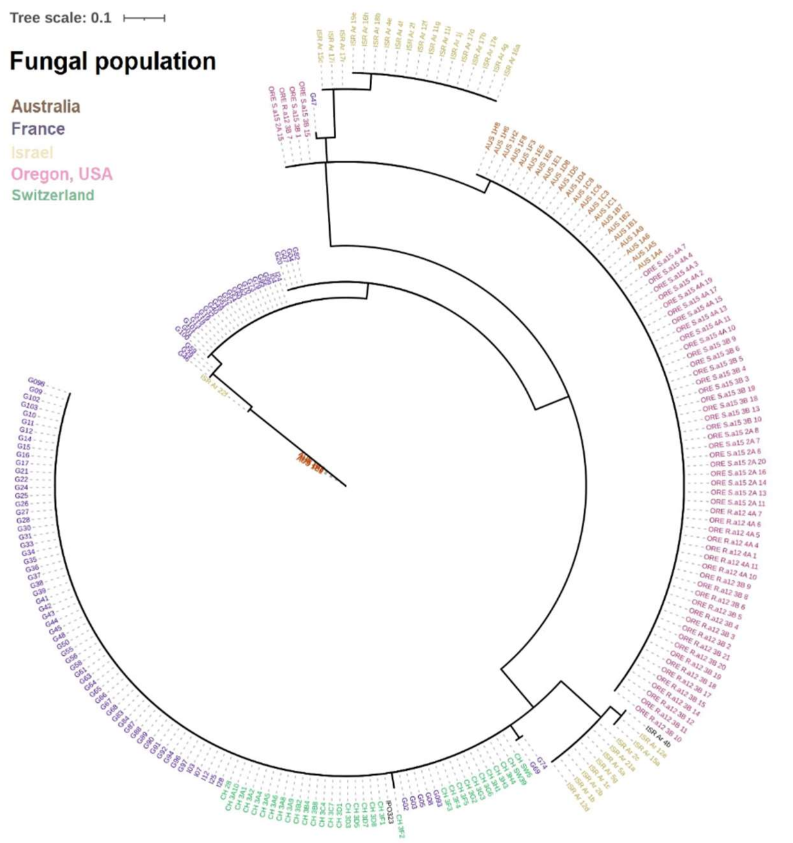

Zt_6_00682

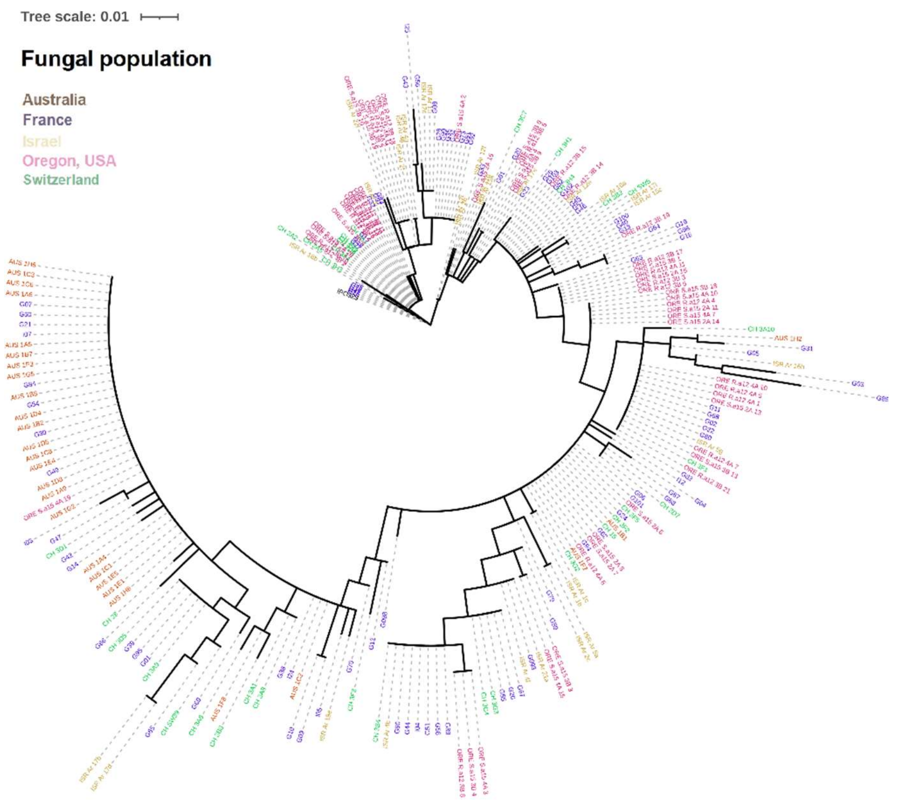

Supplementary Fig. 9 | Phylogenetic tree of *Zt_3_00467* (top) and *Zt_6_00682* (bottom) generated from the protein sequences of the global *Z. tritici* isolates (n=241 and 248, respectively).

Supplementary Table 1 | Descriptive statistics for quantitative pathogenicity traits assessed on differential wheat cultivars.

	Range (%)		Mean (%)		Strandad Deviation		Coefficient of Variation	
**Cultivar**	**PLACN**	**PLACP**	**PLACN**	**PLACP**	**PLACN**	**PLACP**	**PLACN**	**PLACP**
Apache	35 - 100	0.6 - 91.8	93,32	33,74	11,11	18,28	11,91	54,18
Arina	0 - 100	0 - 94.7	73,94	37,1	36,01	26,9	48,7	72,51
Bulgaria-88	0.3 - 100	0 - 54.1	76,12	8,9	28,91	10,85	37,98	121,94
Cadenza	0 - 100	0 - 94.7	69,87	37,24	39,61	31,42	56,69	84,37
Courtot	11.1 - 100	0 - 99.4	79,61	45,33	24,57	28,61	30,86	63,11
Est-Federal	11.1 - 100	1.1 - 96.4	93,15	51,68	16,55	25,88	17,77	50,07
Israel-493	0 - 100	0 -74.2	56,5	18	30,57	16,89	54,11	93,81
Premio	14 - 100	0.1 - 93.7	89,64	44,55	20,9	24,92	23,31	55,93
Salamouni	0 - 68.3	0 - 35.8	15,43	3,82	15,78	5,88	102,29	154
Shafir	0 - 100	0 - 91.7	69,73	32,86	34,85	25,99	49,97	79,09
Tadinia	0 - 100	0 - 71.1	47,31	14,69	35,92	17,39	75,93	118,34
Toisondor	25 - 100	0 - 70	84,89	31,24	15,65	18,08	18,44	57,88

Supplementary Table 2 | Analysis of variance summary table showing the influence of isolate, cultivar and their interaction.

	PLACP					PLACN			
	df	**MS**	***F***	***P***		***df***	**MS**	***F***	***P***
**Isolate**	108	6266	10,73	***		108	11027	18,83	***
**Cultivar**	11	57501	98,52	***		11	128974	220,27	***
**Isolate x Cultivar**	1188	1016	1,74	***		1188	1456	2,48	***
**Error**	2045	584				2045	586		

Supplementary Table 3 | Wheat differentials used to pathotype *Z. tritici* isolates and their known Stb resistance genes indicated in grey (Brown *et al.*, 2015)[1].

Cultivar/*R* gene	***Stb1***	***Stb2***	***Stb3***	***Stb4***	***Stb5***	***Stb6***	***Stb7***	***Stb8***	***Stb9***	***Stb10***	***Stb11***	***Stb12***	***Stb13***	***Stb14***	***Stb15***	***Stb16q***	***StbSm3***	**Reference**
Bulgaria-88	■					■												Adhikari *et al.* 2004c[2]; Chartrain *et al.* 2005b[3]
Veranopolis		■				■												Adhikari *et al.* 2004b[4]; Chartrain *et al.* 2005b[3]
Israel-493			■			■												Adhikari *et al.* 2004b[4]; Chartrain *et al.* 2005b[3]
Tadinia				■		■												Adhikari *et al.* 2004a[5]; Chartrain *et al.* 2005b[3]; Somasco *et al.* 1996[6]
CS-synthetic (69)7D					■	■												Arraiano *et al.* 2001[7]
Cadenza						■			■ ?[1]									Zhong *et al.* 2017[8]; Saintenac *et al.* 2018[9]
Shafir						■												Brading *et al.* 2002[10]
Est.-Federal							■											McCartney *et al.* 2003[11]
M6-synthetic (W-7984)								■										Adhikari *et al.* 2003[12]
Courtot									■									Chartrain *et al.* 2009[13]; Amezrou *et al.* 2023[14]
Kavkaz-K4500						■	■			■		■						Chartrain *et al.* 2005a[15]
TE-9111						■	■				■							Chartrain *et al.* 2005c[16]
Apache				■ ?	■ ?						■							Ghaffary *et al.* 2011[17]
Salamouni													■	■			■	Cowling 2006[18]
Arina						■									■			Arraiano *et al.* 2007[19]; Chartrain *et al.* 2005b[3]
M3-synthetic (W-7976)																■		Ghaffary *et al.* 2012[20]; Saintenac et al. 2021[21]

[1] *Stb9* was detected in the cultivar Cadenza for the first time in this study

Supplementary Table 4 | Functional annotation of candidate pathogenicity genes**.**

Gene name	Gene ID	Protein length	% of Cysteines	SignalP (probability)	# of predicted TMHs	EffectorP (probability)	Conserved protein domain		
							Domain	E-value	Description
	Zt_1_00196	422	2,61		5				
AvrStb9	*Zt_1_00693*	727	1,13	0,99			Peptidase_S41 superfamily	3,99E-02	Peptidase_S41
	Zt_1_01325	95	0,00		2		DPM2	6,29E-32	dolichol phosphate-mannose biosynthesis regulatory
	Zt_1_01674	408	1,23	1,00			TrxB	6,10E-21	Thioredoxin reductase
	Zt_1_01987	475	0,63				cupin_RmlC-like	0,00	cupin_like
MGAMN10	*Zt_2_00163*	506	0,79	1,00			Glyco_hydro_47	0,00	Glycosyl hydrolase family 47
	Zt_2_00165	430	0,00	1,00			Ish1	1,53E-10	Putative stress-responsive nuclear envelope protein
	Zt_2_01230	96	0,00	1,00		0,73			
	Zt_3_00467	126	6,35	1,00		0,86			*SSP - Z. tritici specific*
AvrStb6	-	83	14,63	1,00		0,96			*SSP - Z. tritici specific*
	Zt_5_00252	385	1,04		6				
	Zt_6_00224	69	8,70	1,00		0,85			*SSP - Z. tritici specific*
	Zt_6_00425	329	0,91						
	Zt_6_00682	305	0,33			0,50	Methyltransf_25	5,33E-16	SAM-dependent methyltransferase
	Mycgr3G110052	179	1,12	0,99		0,58			*SSP - Z. tritici specific*
	Zt_9_00069	249	0,00	0,87			adh_short	1,56E-31	Short chain dehydrogenase
	Zt_13_00071	444	1,13						
	Mycgr3G95574	114	4,39	1,00		0,60			*SSP - Z. tritici specific*
	Mycgr3G106276	109	4,59						*Z. tritici specific*

Supplementary Table 4. Continued.

Gene name	Gene ID	PHIB-blast					
		PHI-base ID	Gene	E-value	% Identi-ties	pathogen species	Phenotype
AvrStb9	*Zt_1_00196*	5822	Fgsg1989	8,94E-34	31,35	*Fusarium graminearum*	unaffected pathogenicity
	Zt_1_00693						
	Zt_1_01325						
MGAMN10	*Zt_1_01674*	6471	Trr2	2,41E-17	25,95	*Beauveria bassiana*	reduced virulence
	Zt_1_01987	542	hmgA	1,39E-142	52,86	*Pseudomonas aeruginosa*	increased virulence
	Zt_2_00163	251	msdS/Af msdC	0,00	59,10	*Aspergillus fumigatus*	unaffected pathogenicity
	Zt_2_00165						
AvrStb6	*Zt_2_01230*						
	Zt_3_00467						
	-						
	Zt_5_00252	587	Fgsg4159	4,43E-28	31,27	*Fusarium graminearum*	unaffected pathogenicity
	Zt_6_00224						
	Zt_6_00425						
	Zt_6_00682	3881	LaeA	4,74E-10	24,62	*Aspergillus flavus*	reduced virulence
	Mycgr3G110052						
	Zt_9_00069	1992	GzZC37	3,42E-48	55,53	*Fusarium graminearum*	unaffected pathogenicity
	Zt_13_00071						
	Mycgr3G95574						
	Mycgr3G106276						

Supplementary Table 5 | Diversity statistics of pathogenicity genes.

	Gene presence (%)	Segr. Sites	Haplo-types	Haplotype diversity	π	Tajima D		Fu & Li D		Fu & Li F		ZnS (LD)	GC content (%)	TE distance (Kb)
Mean	97,04	104,4561	47,737	0,8376	0,0196	-0,0340		-1,1770		-0,7986		0,1546	0,5425	15,25
Median	100,00	65,00	39,00	0,90	0,02	0,88		-0,26		0,04		0,16	0,55	4,15
Avrstb6	100,00	65	18	0,7750	0,0255	-1,2017	n.s.	0,5553	n.s.	-0,2092	n.s.	0,3104	0,5448	2,07
AvrStb9	100,00	81	28	0,8362	0,0051	-0,9460	n.s.	0,4286	n.s.	-0,1746	n.s.	0,1629	0,5392	2,93
Mycgr3G106276	100,00	28	35	0,9086	0,0230	1,0792	n.s.	0,5038	n.s.	0,8379	n.s.	0,1615	0,5146	4,15
Mycgr3G110052	51,46	145	10	0,5806	0,1182	1,2962	n.s.	2,0417	**	2,0673	**	0,5112	0,4457	2,23
Mycgr3G95574	100,00	48	40	0,9098	0,0323	-0,6859	n.s.	1,3557	n.s.	0,6459	n.s.	0,1696	0,5904	3,55
Zt_1_00196	100,00	31	29	0,6674	0,0015	-2,0907	*	-5,5494	**	-4,8307	**	0,0355	0,5492	0,48
Zt_1_01325	100,00	17	13	0,5189	0,0108	0,8775	n.s.	-1,3548	n.s.	-0,6079	n.s.	0,3324	0,5492	2,98
Zt_1_01674	100,00	75	68	0,9743	0,0105	-0,1741	n.s.	-1,9473	#	-1,3726	n.s.	0,0510	0,5487	14,59
Zt_1_01987	100,00	125	55	0,9271	0,0300	2,2148	*	1,1640	n.s.	1,9050	*	0,1187	0,5816	17,88
Zt_13_00071	98,06	96	43	0,9061	0,0293	1,4282	n.s.	-1,0453	n.s.	0,0431	n.s.	0,1627	0,5508	16,55
Zt_2_00163	100,00	145	92	1,0000	0,0322	2,4158	*	0,7520	n.s.	1,7482	*	0,0534	0,5541	21,96
Zt_2_00165	100,00	50	45	0,8247	0,0133	-0,1118	n.s.	-2,1126	n.s.	-1,5075	n.s.	0,0830	0,5761	17,21
Zt_2_01230	100,00	17	22	0,8093	0,0047	-1,1630	n.s.	-4,3619	**	-3,6519	**	0,0199	0,5396	0,18
Zt_3_00467	97,09	33	16	0,8358	0,0145	2,4201	*	1,0260	n.s.	1,9408	#	0,3217	0,5489	0,04
Zt_5_00252	100,00	295	52	0,9044	0,0520	1,7580	#	1,2515	n.s.	1,7403	*	0,2805	0,5343	36,53
Zt_6_00224	99,03	22	24	0,8515	0,0050	-0,5148	n.s.	-2,7348	**	-2,1360	*	0,0513	0,5568	5,19
Zt_6_00425	100,00	156	97	0,9989	0,0495	1,0488	n.s.	-0,2640	n.s.	0,4079	n.s.	0,0906	0,5856	10,19
Zt_6_00682	100,00	43	39	0,9299	0,0212	1,1329	n.s.	-0,6695	n.s.	0,0652	n.s.	0,2225	0,6068	4,39
Zt_9_00069	98,06	95	51	0,8980	0,0128	-1,6910	#	-2,5183	*	-2,4677	*	0,1406	0,5606	7,13

n.s. not significant

# P<0.1

** P<0.05*

*** P<0.01*

**** P<0.001*

Supplementary Table 6 | Summary of recombination and selection analyses of pathogenicity genes.

	Recombination		codeML							
					M8 model parameters					
Gene ID	Breakpoints[a]	Events[b]	M1a vs M2a	M7 vs M8	dN/dS[c]	lnL[d]	ω[e]	p[f]	PSS[g]	% of positively selected sites
AvrStb6	2	-	<.001	<.001	2,84	-726,623	17,129	0,147	10 (9)	10,98
AvrStb9	2	1	<.001	<.001	1,04	-3137,804	15,137	0,069	12 (5)	0,69
Mycgr3G106276	1	-	<.001	<.001	1,18	-775,735	10,420	0,092	6 (5)	4,59
Mycgr3G110052	2	2	Presence/absence polymorphism + partial deletion							NA
Mycgr3G95574	-	11	<.001	<.001	0,34	-1243,004	4,879	0,048	3 (2)	1,75
Zt_1_00196	-	-	*NS*	*NS*	0,12	-1942,141	7,398	0,010	none	NA
Zt_1_01325	-	-	*NS*	*NS*	1,00	-398,754	1,000	1,000	none	NA
Zt_1_01674	2	-	<.001	<.001	0,12	-2623,182	4,828	0,016	4 (3)	0,74
Zt_1_01987	5	17	<.001	<.001	0,04	-5452,238	1,940	0,017	2 (2)	0,42
Zt_13_00071	1	12	<.001	<.001	0,33	-4221,104	2,907	0,084	9 (3)	0,68
Zt_2_00163	2	10	*NS*	*NS*	0,25	-650,262	1,000	0,000	none	NA
Zt_2_00165	1	4	<.001	<.001	0,17	-3637,802	4,708	0,031	9 (7)	1,63
Zt_2_01230	-	-	*NS*	*NS*	0,17	-450,211	1,000	0,000	none	NA
Zt_3_00467	2	1	<.001	<.001	7,03	-588,796	50,813	0,127	9 (6)	4,76
Zt_5_00252	2	6	<.001	<.001	0,41	-3131,581	3,366	0,102	13 (5)	1,30
Zt_6_00224	-	-	<.001	<.001	0,87	-488,880	7,113	0,123	3 (2)	2,90
Zt_6_00425	2	12	<.001	<.001	0,31	-8130,354	2,689	0,091	27 (24)	7,29
Zt_6_00682	1	1	<.001	<.001	0,13	-2904,540	7,783	0,010	3 (3)	0,98
Zt_9_00069	-	2	*NS*	*NS*	0,11	-1950,595	1,000	0,070	none	NA

[a]Number of recomination breakpoints detected by GARD

[b]Number of recombination event detected by at least 5 algorithms in RDP

[c]non-synonymous/synonymous substitution ratios across all gene tree under the model M8

[d]lnL the likelihood of the experimental model

[e]dN/dS ratio estimated in the model parameters

[f]the portion of positively selected sites estimated in the model parameters

[g]Positiveley selected sites with a NEB posterior probability > 0.95. Sites with a BEB probability > 0.95 are under parenthesis.

Supplementary Table 7 | List of primers used for functional studies.

No.	Primer name	Primer sequence	Amplification
		Zt_6_00682	
1	pNOV_for	ATGACGCGGGACAAG	Vector, KO and ectopic transformants validation
2	pNOV_rev	TAACACATTGCGGATACG	Vector, KO and ectopic transformants validation
3	Hygro_for	tgatattgaaggagcat	cloning KO transformants
4	Hygro_rev	TCTATTCCTTTGCCC	cloning KO transformants
19	Hygro_rev2	GGGATCAGCAATCGC	KO vector validation
20	Hygro_for2	CTGCCTGAAACCGAAC	KO vector validation
23	pNOV_inv_for	ATTCGAATCCGGAGC	cloning KO
24	pNOV_inv_rev	ATGGTCGACTCTAGAGGATC	cloning KO
47	5’ 3D7 IPO for	GGATCCTCTAGAGTCGACCATCATGATGACACCTCAGGCGTC	cloning KO
48	5’ 3D7 IPO rev	CCAAAAAATGCTCCTTCAATATCAGTTGAAGTCTTTTTGATGGTGGTA	cloning KO
49	3’ 3D7 for	CGTCCGAGGGCAAAGGAATAGATGGTTGGAAGTGGTGCTGATG	cloning KO
51	3’ 3D7 IPO rev	TGCGGCCGCTCCGGATTCGAATATCCGCGAGGAGATGGAGAATA	cloning KO
72	Gibsecto_3D7_for	GAATTCGAGCTCGGTACGTTGGTGGATGTGATCTGTG	ectopic cloning
73	Gibsecto_3D7_rev	AATGCTCCTTCAATATCAATCAAAACAGAGCTCTCGTG	ectopic cloning
74	Gibsecto_3D1_for	GAATTCGAGCTCGGTACGTTGGTGGATGTGATCTGTG	ectopic cloning
75	Gibsecto_3D1_rev	AATGCTCCTTCAATATCAACTACGAAAGTCAAAACGGA	ectopic cloning
76	Seq 3D1 3D7 for	CTAGTCCATCTCGCCATTC	validation ectopic vector and ectopic transformants
77	Seq 3D1 3D7 rev	CCCTCACCACGAAACTC	validation ectopic vector and ectopic transformants
78	Seq 3D1 3D7 for 2	ATCCGCAAAGTCGTCG	validation vecteur ectopique
79	Seq 3D1 3D7 for 3	CTTCAGGATGCGAGAC	validation vecteur ectopique
94	ValidKO_3D7IPO_F	TTGAGTTTCGTGGTG	Validation KO transformants
95	ValidKO_3D7IPO_R	GGTCATGCATACATTG	Validation KO transformants
46	pNOV_sulf_rev	CGCCTGGACGACTAAAC	validation ectopic vector
117	Pnov_for BIS	CAGCGGCCATTTAAATC	validation ectopic transformants
		Zt_3_00467	
ASV10p	Zt_3_00467_AVR_IF_F1	GCGCGCCGAATTCGAGCTCATTAACCGCTCTTGGTCGCT	Ectopic expression of Zt_3_00467 3D7 and IPO10273 alleles
ASV11p	Zt_3_00467_AVR_IF_R1	CCAACATGGTGGAGTGAGGGTCCAGGAACAACCTTGCAC	Ectopic expression of Zt_3_00467 3D7 and IPO10273 alleles

Supplementary References

1. Brown, J. K. M., Chartrain, L., Lasserre-Zuber, P., & Saintenac, C. Genetics of resistance to *Zymoseptoria tritici* and applications to wheat breeding. *Fungal Genet. Biol.* **79**, 33–41 (2015). https://doi.org/10.1016/j.fgb.2015.04.017
2. Adhikari, T. B., et al. Molecular mapping of *Stb1*, a potentially durable gene for resistance to septoria tritici blotch in wheat. *Theor. Appl. Genet.* **109**, 944-953 (2004c). https://doi.org/10.1007/s00122-004-1709-6
3. Chartrain, L., Brading, P. A., & Brown, J. K. M. Presence of the *Stb6* gene for resistance to septoria tritici blotch (*Mycosphaerella graminicola*) in cultivars used in wheat-breeding programmes worldwide. *Plant Pathol.* **54**, 134-143 (2005b). https://doi.org/10.1111/j.1365-3059.2005.01164.x
4. Adhikari, T. B., Wallwork, H. & Goodwin, S. B. Microsatellite markers linked to the *Stb2* and *Stb3* genes for resistance to septoria tritici blotch in wheat. *Crop Sci.* **44**, 1403-1411 (2004b). https://doi.org/10.2135/cropsci2004.1403
5. Adhikari, T. B., et al. Molecular mapping of the *Stb4* gene for resistance to septoria tritici blotch in wheat. *Phytopathology* **94**, 1198-1206 (2004a). https://doi.org/10.1094/PHYTO.2004.94.11.1198
6. Somasco, O. A., Qualset, C. O., & Gilchrist, D. G. Single-gene resistance to Septoria tritici blotch in the spring wheat cultivar 'Tadinia'. *Plant Breed.* **115**, 261-267 (1996). https://doi.org/10.1111/j.1439-0523.1996.tb00914.x
7. Arraiano, L. S., Worland, A.J., Ellerbrook, C., & Brown, J.K.M. Chromosomal location of a gene for resistance to septoria tritici blotch (*Mycosphaerella graminicola*) in the hexaploid wheat 'Synthetic 6x'. *Theor. Appl. Genet.* **103**, 758-764 (2001). https://doi.org/10.1007/s001220100668
8. Zhong, Z., et al. A small secreted protein in *Zymoseptoria tritici* is responsible for avirulence on wheat cultivars carrying the *Stb6* resistance gene. *New Phytol.* **214**, 619–631 (2017). https://doi.org/10.1111/nph.14434
9. Saintenac, C., et al. Wheat receptor-kinase-like protein Stb6 controls gene-for-gene resistance to fungal pathogen *Zymoseptoria tritici*. *Nat. Genet.* **50**, 368–374 (2018). https://doi.org/10.1038/s41588-018-0051-x
10. Brading, P. A., Verstappen, E. C. P., Kema, G. H. J., & Brown, J. K. M. A gene-for-gene relationship between wheat and *Mycosphaerella graminicola*, the Septoria tritici blotch pathogen. *Phytopathology* **92**, 439–445 (2002). https://doi.org/10.1094/PHYTO.2002.92.4.439
11. McCartney, C. A., Brûlé-Babel, A. L., Lamari, L., & Somers, D. J. Chromosomal location of a race-specific resistance gene to *Mycosphaerella graminicola* in the spring wheat ST6. *Theor. Appl. Genet.* **107,** 1181-1186 (2003). https://doi.org/10.1007/s00122-003-1359-0
12. Adhikari, T. B., Anderson, J. M., & Goodwin S. B. Identification and molecular mapping of a gene in wheat conferring resistance to *Mycosphaerella graminicola*. *Phytopathology* **93**, 1158-1164 (2003). https://doi.org/10.1094/PHYTO.2003.93.9.1158
13. Chartrain, L., Sourdille, P., Bernard, M., & Brown, J. K. M. Identification and location of *Stb9*, a gene for resistance to septoria tritici blotch in wheat cultivars Courtot and Tonic. *Plant Pathol.* **58**, 547-555 (2009). https://doi.org/10.1111/j.1365-3059.2008.02013.x
14. Amezrou, R., et al. A secreted protease-like protein in *Zymoseptoria tritici* is responsible for avirulence on *Stb9* resistance gene in wheat. *PLoS Pathog.* **19**, e1011376 (2023). https://doi.org/10.1371/journal.ppat.1011376
15. Chartrain, L., Berry, S. T., & Brown, J. K. M. Resistance of wheat line Kavkaz-K4500 L.6.A.4 to septoria tritici blotch controlled by isolate-specific resistance genes. *Phytopathology* **95,** 664-671 (2005a). https://doi.org/10.1094/PHYTO-95-0664

16. Chartrain, L., et al. Genetics of resistance to septoria tritici blotch in the Portuguese wheat breeding line TE 9111. *Theor. Appl. Genet.* **110**, 1138-1144 (2005c). https://doi.org/10.1007/s00122-005-1945-4

17. Ghaffary, S. M. T., et al. Genetic analysis of resistance to septoria tritici blotch in the French winter wheat cultivars Balance and Apache. *Theor. Appl. Genet.* **123**: 741-754 (2011). https://doi.org/10.1007/s00122-011-1623-7

18. Cowling, S.G. Identification and mapping of host resistance genes to septoria tritici blotch of wheat. (Ph.D. thesis, University of Manitoba, 2006). http://hdl.handle.net/1993/20699

19. Arraiano, L. S., et al. A gene in European wheat cultivars for resistance to an African isolate of *Mycosphaerella graminicola*. *Plant Pathol.* **56,** 73-78 (2007). https://doi.org/10.1111/j.1365-3059.2006.01499.x

20. Ghaffary, S. M. T., et al. New broad-spectrum resistance to septoria tritici blotch derived from synthetic hexaploid wheat. *Theor. Appl. Genet.* **124**, 125-142 (2012). https://doi.org/10.1007/s00122-011-1692-7

21. Saintenac, C., et al. A wheat cysteine-rich receptor-like kinase confers broad-spectrum resistance against Septoria tritici blotch. *Nat. Commun.* **12**, 433 (2021). https://doi.org/10.1038/s41467-020-20685-0
