# Supplementary material for: Quantitative pathogenicity and host adaptation in a fungal plant pathogen revealed by whole-genome sequencing
Source: Nat Commun. 2024 Mar 2;15:1933. doi: 10.1038/s41467-024-46191-1 (PMC10908820; doi:10.1038/s41467-024-46191-1)
Supplement: Supplementary file 3 — Reporting Summary [file 41467_2024_46191_MOESM3_ESM.pdf]

nature portfolio

Corresponding author(s): Thierry C. MARCEL

Last updated by author(s): 2023/12/18

Reporting Summary

Nature Portfolio wishes to improve the reproducibility of the work that we publish. This form provides structure for consistency and transparency in reporting. For further information on Nature Portfolio policies, see our Editorial Policies and the Editorial Policy Checklist.

Statistics

For all statistical analyses, confirm that the following items are present in the figure legend, table legend, main text, or Methods section.

n/a	Confirmed	
☐	☒	The exact sample size (*n*) for each experimental group/condition, given as a discrete number and unit of measurement
☐	☒	A statement on whether measurements were taken from distinct samples or whether the same sample was measured repeatedly
☐	☒	The statistical test(s) used AND whether they are one- or two-sided<br>*Only common tests should be described solely by name; describe more complex techniques in the Methods section.*
☐	☒	A description of all covariates tested
☐	☒	A description of any assumptions or corrections, such as tests of normality and adjustment for multiple comparisons
☐	☒	A full description of the statistical parameters including central tendency (e.g. means) or other basic estimates (e.g. regression coefficient) AND variation (e.g. standard deviation) or associated estimates of uncertainty (e.g. confidence intervals)
☐	☒	For null hypothesis testing, the test statistic (e.g. *F*, *t*, *r*) with confidence intervals, effect sizes, degrees of freedom and *P* value noted<br>*Give P values as exact values whenever suitable.*
☐	☒	For Bayesian analysis, information on the choice of priors and Markov chain Monte Carlo settings
☒	☐	For hierarchical and complex designs, identification of the appropriate level for tests and full reporting of outcomes
☐	☒	Estimates of effect sizes (e.g. Cohen's *d*, Pearson's *r*), indicating how they were calculated

Our web collection on statistics for biologists contains articles on many of the points above.

Software and code

Policy information about availability of computer code

Data collection	Candidate pathogenicity genes were blasted against the following databases: PFAM 35.0 <http://pfam.xfam.org/>; PHI-base <http://www.phi-base.org/>.
Data analysis	All the software and open source codes used to analyse the data are appropriately provided in the Methods section of the manuscript. The list of software and packages used for the analyses comprises: BWA v0.7.7; Samtools v0.1.19; Samtools v1.10; Picard tools v1.106; Freebayes v0.9; RepeatMasker Open-4.0; REPET v2.5; PLINK v1.9; PopLDdecay v3.40; STRUCTURE v2.2; GAPIT v3.0 [EMMA algorithm]; GEC; Haploview v4.2; SignalP 3.0; TMHMM 2.0; EffectorP 3.0; Cuffdiff v.2.2.1; SPAdes v.3.14.1; MAFFT v7.464; JalView v2.11.1; trimAL v1.2; DNAsp v.6; bedtools v2.29.2; RDP4.101 [RAxML algorithm]; GARD; PAML v4.9.We didn't use any custom codes in this manuscript.

For manuscripts utilizing custom algorithms or software that are central to the research but not yet described in published literature, software must be made available to editors and reviewers. We strongly encourage code deposition in a community repository (e.g. GitHub). See the Nature Portfolio guidelines for submitting code & software for further information.

Data

Policy information about availability of data

All manuscripts must include a data availability statement. This statement should provide the following information, where applicable:

- Accession codes, unique identifiers, or web links for publicly available datasets
- A description of any restrictions on data availability
- For clinical datasets or third party data, please ensure that the statement adheres to our policy

The sequence and annotation of Z. tritici IPO323 reference genome are available from DOE Joint Genome Institute website <https://mycocosm.jgi.doe.gov/Zymtr1/Zymtr1.home.html>. Sequence data of the 103 French isolates are deposited at the NCBI Sequence Read Archive under the accession number PRJNA777581. Sequence data for the global isolates are available at the NCBI Sequence Read Archive under the accession number PRJNA327615. RNAseq data were provided by Robert King and Jason Rudd at Rothamsted Research (Harpenden, United Kingdom).

Research involving human participants, their data, or biological material

Policy information about studies with human participants or human data. See also policy information about sex, gender (identity/presentation), and sexual orientation and race, ethnicity and racism.

Reporting on sex and gender	Not applicable
Reporting on race, ethnicity, or other socially relevant groupings	Not applicable
Population characteristics	Not applicable
Recruitment	Not applicable
Ethics oversight	Not applicable

Note that full information on the approval of the study protocol must also be provided in the manuscript.

Field-specific reporting

Please select the one below that is the best fit for your research. If you are not sure, read the appropriate sections before making your selection.

☒ Life sciences ☐ Behavioural & social sciences ☐ Ecological, evolutionary & environmental sciences

For a reference copy of the document with all sections, see nature.com/documents/nr-reporting-summary-flat.pdf

Life sciences study design

All studies must disclose on these points even when the disclosure is negative.

Sample size	Sample size of the Z. tritici population analyzed (n=103) was determined according to our phenotyping capacity of individual fungal isolates: we phenotyped 103 isolates on 12 cultivars in 3 replications with 3 leaves per replication, which represent more than 11 000 individual leaves being inoculated and observed. A reduced sample size will have for consequence a reduced statistical power to detect associations through GWAS, but we still were able to detect significant associations. Filtering parameters applied on the SNP matrix were adjusted according to this sample size.
Data exclusions	A SNP matrix was obtained after mapping sequences of the 103 Z. trititi isolates on the reference genome sequence of the isolate IPO-323. SNP with more than 50% missing data or a Minor Allele Frequency (MAF) lower than 10% were excluded from the analysis. This filtering allows to avoid biais in GWAS analyses, and tresholds were decided according to the sample size of the experiment (n=103 isolates).
Replication	All plant cultivar-fungal isolate interactions were evaluated in 3 replications with 3 leaves per replication (nine leaves per interaction). All attempts at replication were successful.
Randomization	The repartition of fungal isolates in the growth chamber was randomized between each replication. The same growth chamber was used throughout the phenotyping experiments.
Blinding	The name of the isolates was coded during the phenotyping experiments.

Reporting for specific materials, systems and methods

We require information from authors about some types of materials, experimental systems and methods used in many studies. Here, indicate whether each material, system or method listed is relevant to your study. If you are not sure if a list item applies to your research, read the appropriate section before selecting a response.

Materials & experimental systems

n/a	Involved in the study
☒	☐ Antibodies
☒	☐ Eukaryotic cell lines
☒	☐ Palaeontology and archaeology
☒	☐ Animals and other organisms
☒	☐ Clinical data
☒	☐ Dual use research of concern
☐	☒ Plants

Methods

n/a	Involved in the study
☒	☐ ChIP-seq
☒	☐ Flow cytometry
☒	☐ MRI-based neuroimaging

Dual use research of concern

Policy information about dual use research of concern

Hazards

Could the accidental, deliberate or reckless misuse of agents or technologies generated in the work, or the application of information presented in the manuscript, pose a threat to:

No	Yes
☒	☐ Public health
☒	☐ National security
☐	☒ Crops and/or livestock
☒	☐ Ecosystems
☒	☐ Any other significant area

Hazards: The discovery of a gene from the fungal pathogen Zymoseptoria tritici, the deletion of which enhances pathogenicity on wheat. No agent is subject to oversight.

For examples of agents subject to oversight, see the United States Government Policy for Institutional Oversight of Life Sciences Dual Use Research of Concern.

Experiments of concern

Does the work involve any of these experiments of concern:

No	Yes
☒	☐ Demonstrate how to render a vaccine ineffective
☒	☐ Confer resistance to therapeutically useful antibiotics or antiviral agents
☐	☒ Enhance the virulence of a pathogen or render a nonpathogen virulent
☒	☐ Increase transmissibility of a pathogen
☒	☐ Alter the host range of a pathogen
☒	☐ Enable evasion of diagnostic/detection modalities
☒	☐ Enable the weaponization of a biological agent or toxin
☒	☐ Any other potentially harmful combination of experiments and agents

Precautions and benefits

Biosecurity precautions	Bio-containment facilities were used to produce the transgenic strains of Z. tritici and evaluate their pathogenicity on wheat cultivars.
Biosecurity oversight	None.
Benefits	Fungal diseases can have a significant impact on agriculture, leading to crop losses and affecting global food security. Therefore, comprehending the genetic basis of fungal pathogenicity is fundamental for developing robust strategies to counter fungal epidemics. This involves early detection and disease surveillance, enhanced management of resistance genes, and a deep understanding of infection mechanisms to implement new and effective control strategies.
Communication benefits	The risk of releasing the transgenic Z. tritici strains in nature is extremely limited as biocontainment facilities are used and prophylaxy measures taken to ensure they could not come into contact with wheat crop.
