# Supplementary material for: Quantitative pathogenicity and host adaptation in a fungal plant pathogen revealed by whole-genome sequencing
Source: Nat Commun. 2024 Mar 2;15:1933. doi: 10.1038/s41467-024-46191-1 (PMC10908820; doi:10.1038/s41467-024-46191-1)
Supplement: Supplementary file 4 — Description of Additional Supplementary Files [file 41467_2024_46191_MOESM4_ESM.pdf]

<u>Description of Additional Supplementary Files</u>

File Name: Supplementary Data 1

Description: Summary of significant associations identified by GWAS using 103 Z. tritici isolates and quantitative pathogenicity phenotypes on wheat differential cultivars.

File Name: Supplementary Data 2

Description: Gene expression levels and differential expression analysis (log2 fold change compared to the culture medium CDB) at five time points during infection on wheat.
